# Active Aging: Exploration into Self-Ratings of “Being Active,” Out-of-Home Physical Activity, and Participation among Older Australian Adults Living in Four Different Settings

**DOI:** 10.1155/2015/501823

**Published:** 2015-08-05

**Authors:** Rosemary L. Aird, Laurie Buys

**Affiliations:** School of Design, Queensland University of Technology, Brisbane, QLD 4001, Australia

## Abstract

We examined whether self-ratings of “being active” among older people living in four different settings (major city high and lower density suburbs, a regional city, and a rural area) were associated with out-of-home participation and outdoor physical activity. A mixed-methods approach (survey, travel diary, and GPS tracking over a one-week period) was used to gather data from 48 individuals aged over 55 years. Self-ratings of “being active” were found to be positively correlated with the number of days older people spent time away from home but unrelated to time traveled by active means (walking and biking). No significant differences in active travel were found between the four study locations, despite differences in their respective built environments. The findings suggest that additional strategies to the creation of “age-friendly” environments are needed if older people are to increase their levels of outdoor physical activity. “Active aging” promotion campaigns may need to explicitly identify the benefits of walking outdoors to ambulatory older people as a means of maintaining their overall health, functional ability, and participation within society in the long-term and also encourage the development of community-based programs in order to facilitate regular walking for this group.

## 1. Introduction

Together, the World Health Organization's [1] policy framework for active aging and its guide for the development of “age-friendly” cities [2] have set a policy and research agenda in which the built environment is conceived to play a crucial role as a facilitator or inhibitor of older people's capacity to age “actively.” This theoretical standpoint assumes that built environments which are conducive to the outdoor mobility of older people will help provide them with ongoing opportunities for both participation within society and maintenance of their health through the gaining of physical exercise as they move around their neighborhoods. It remains unclear, however, whether or not older people's self-assessments of “being active” are linked to either their levels of participation within society or their engagement in outdoor physical activity, and, to date, there is no convincing evidence of a direct relationship between the built environment and older people's walking behavior.

“Active aging” is defined by the WHO [1, page 12] as “the process of optimizing opportunities for health, participation, and security in order to enhance quality of life as people age.” This concept presents both opportunities and challenges to policy makers and researchers alike. In terms of policy, active aging has been acknowledged as providing a sound basis for responding to the challenges posed by population aging in industrialized nations by linking a number of key policy domains including employment, pensions, retirement, health, and citizenship [3, page 121]. Yet within Europe, for example, the focus on active aging has so far been largely limited to a “crude reduction in terms of working longer” [4, page S117]. Walker [3, page 124] noted more than a decade ago that active aging “does not amount to a coherent strategy and is sometimes just a slogan used to cover anything that seems to fit under it.” More recently, he has stressed that this concept is more of an aspiration for both individuals and collectivities than it is a process that exists in practice [4, page S126]. The usefulness of the active aging concept for policy makers aiming to comprehensively address issues associated with the growing aging population will necessarily rest on their capacity to devise a coherent set of strategies that effectively deal with all of the three key determinants of active aging identified by the WHO—participation, health, and security. The sheer scope of this concept makes this a difficult task.

One key opportunity presented to researchers by the development of the active aging concept is its potential to act as a driver for greater attention on the older person-macroenvironment relationship. Indeed, there has been unprecedented research interest in this area since the release of the WHO's [1] policy framework; prior to the early 2000s, it was the microenvironments of older people which predominated as the main focus of investigations into the older person-environment relationship (see [5]). Nevertheless, the complex, multifaceted nature of the active aging concept is problematic, especially since it effectively encompasses dimensions covered by a number of earlier concepts. These include “successful ageing” which Walker [3] traces back to the early 1960s (a concept emphasizing the importance of maintaining activity patterns and values of middle age into old age) and “productive aging” which emerged in the 1980s (mostly focused on removing barriers to older persons engaging in work—paid or unpaid—and thereby extending their productive capacity beyond retirement age). Walker [3, page 123-124] notes that “active aging” emerged in the 1990s, giving recognition to both the connection between activity and health and the importance of “healthy aging.” His account of the development of the active aging concept clearly demonstrates that active aging is much broader in scope than all of these earlier concepts. Nevertheless, a review of empirical literature recently undertaken by Hung and colleagues [6] grouped “active aging” with “successful,” “productive,” “positive,” and “robust” aging as cognates of “healthy aging”—despite the fact that the active aging concept encompasses more dimensions than any of these other concepts (including continued participation in society; the maximization of social, mental, and physical health; the maintenance of dignity, self-efficacy, and human rights; and the creation of age-friendly physical environments to facilitate autonomy and independence) (see [3, 7]). Coherency of the accumulating evidence on active aging may well be undermined by both the overlap between this concept and its predecessors and the treatment of them as interchangeable terms. It is also noteworthy that “successful aging” has now been a focus of research for around half a century and there is still no widely agreed definition for this term or consistency between studies in the measures used to investigate this subject area, and the first rigorous analysis of measures was only undertaken recently (see [8]). There would seem to be much greater potential for disagreement about how “active aging” is operationally defined by researchers given the broad scope of this concept. This is not only an issue for the research community. If active aging objectives are to be met, it would seem crucial that laypersons ascribe meanings to this term that are in line with the WHO's [1] policy framework and fully grasp the nature and importance of all of the constituents of active aging as laid out in this framework.

To date, only a few studies have explored older people's perceptions of active aging. Research undertaken in the United Kingdom by Bowling [7] found this term was most commonly conceived by a sample of older people aged 60 years and over, to involve having/maintaining physical health and functioning (43%), leisure and social activities (34%), mental functioning and activity (18%), and social relationships and contacts (15%). Comparison with academic conceptualizations of active aging showed that there was overlap between lay perceptions of the constituents of active ageing and those used in theoretical models within the literature, although the latter entailed additional components (productivity, empowerment, human rights, and dignity) to those identified by laypersons. Multiple regression analyses revealed that health status, longstanding illness, and quality of life (all based on self-report) explained 41% of the variance in self-ratings of active aging, while sociodemographic and economic variables were not significantly associated with this outcome [7, page 298]. Comparison of data gathered on the perceived constituents of active aging in this particular study with data obtained from an earlier survey of older people's perceptions of successful aging, also administered in the United Kingdom but with adults aged 50 years and over (for further details see [9]), showed that there was considerable overlap in the meanings ascribed to “active” and “successful” aging. A later study by Bowling [10] compared the perceptions of minority ethnic groups with those of the general population in the United Kingdom, revealing that ethnically diverse respondents were less likely to define active aging in terms of good physical health and fitness (and exercise to promote these two aims) and also less likely to rate themselves as aging actively than respondents within the general population. From Bowling's [7] research, it would seem that individual factors such as health status and longstanding illness impact on older people's self-assessments of aging actively, but we currently have little understanding of whether or not there is any relationship between these self-assessments and the actual behaviors of older persons with respect to their actual levels of participation and physical activity outside of the home domain.

Older people's participation in the wider community necessarily relies on their capacity for remaining mobile in their out-of-home environments. The accumulated body of evidence suggests that individual factors as well as multiple aspects of the built environment affect older people's potential out-of-home mobility and thus their capacity to participate in society. Driving status [11, 12], quality and availability of public transport [13, 14], and numerous other features of the built environment (see [15, 16]) are all implicated in this regard. Walkable environments are particularly important, given that neighborhood walking increases not only older people's opportunities for physical exercise but also social interaction [17]. Engagement in outdoor physical activity by older people is crucial to them remaining ambulatory and also provides numerous other health benefits [18].

In the United States, lack of physical activity among older people has been described as a major public health issue; the release of Surgeon-General's Report in 1996 indicated that less than one-third of adults aged 65 years and over met health recommendations of moderate intensity exercise (e.g., brisk walking) for 30 minutes, five or more days per week [19, S267]. More recent research conducted in New Jersey shows a similar pattern, with just 28.6 percent of 50- to 74-year-olds being found to meet exercise recommendations [20]. The proportion of older people who meet exercise guidelines has been shown to be highly variable, however, both within and between different countries. A systematic review of studies investigating physical activity among older adults (limited to those studies which reported their findings in terms of older people meeting exercise guidelines) revealed that this proportion ranges between 2.4 and 83.0 percent, with the majority of the 53 studies reviewed being found to report proportions of between 20 and 60 percent [21]. Only 6 of these studies (undertaken in the United States, Australia, the United Kingdom, Brazil, China, Canada, New Zealand, Columbia, South Africa, Greece, Cyprus, Sweden, and Switzerland) used an objective measure of physical activity (i.e., accelerometer), with the remainder being based on self-reported activity.

Research efforts over the past decade have provided insight into many features of the built environment that encourage or constrain older people's walking decisions. Yet, the extent to which older people's walking behavior actually differs as a function of the particular built environment in which they live remains unclear. Much of the research literature related to the built environment and walking activity is based on random samples, with findings being largely representative of those living in urbanized areas (i.e., similar built environments) [22]. In addition, the vast majority of available studies of walking behavior among older people are based on self-report, making the findings subject to bias (e.g., participant recall and selection of socially acceptable responses). A recent review of empirical literature that examined mobility or disability among older people and also utilized objective measures of the built environment [15] identified 17 studies published between 1990 and 2010 that met these criteria. Of these, 14 studies investigated walking behavior, with all but one [23] being based on self-reports of walking. Rosso et al. [15] concluded that although evidence of a direct relationship between the built environment and elderly walking behavior was lacking, of all of the features of the built environment investigated, high density of intersections, street and traffic conditions, proximity to destinations, and green space were the factors most likely to have a direct effect on older people's out-of-home mobility. Nevertheless, a study of the consumption patterns and mode of travel used by older people (via GPS tracking) for accessing goods and services within two different countries (suburbs of Canada and France) showed that older people in both locations were highly reliant on travel by motor vehicle, despite substantial differences in the built environment and availability of public transport in the two study sites [24]. This raises questions about the assumption that levels of walking outdoors among the older segment of the population will naturally follow modifications to the built environment that make them more “age-friendly,” without a shift away from sedentary lifestyles and a change in the propensity of the elderly to rely predominantly on motor vehicles for movement outside of the home domain.

Older people have identified particular urban design features such as green and open spaces [25, 26] as being conducive to walking. Other features like poorly maintained pedestrian infrastructure and traffic, however, heighten their fears about personal safety [27]. Evidence suggests that these fears about safety are well grounded, given that most falls occur outside of the home domain with the majority taking place on curbs, sidewalks, and streets [28] and that older people are the most likely group to present at hospital for injuries sustained from pedestrian-cyclist collisions [29]. Unfortunately, however, fear of moving outdoors also increases older people's risk of diminished capacity to walk outdoors in the longer term. Longitudinal research has shown that older people who fear walking outdoors are 4.6 times more likely than those without this fear to develop difficulties in walking a distance of 0.5 kms [30]. Older people's personal expectations about aging also influence their walking behavior. A pilot test of a behavioral intervention aimed at altering belief in the inevitability of becoming sedentary as a consequence of the aging process demonstrated that older people not only increased their walking after intervention but also experienced multiple other benefits including ease in performing daily life tasks and improvements in mental health-related quality of life, pain, energy, and quality of sleep [31].

Meeting the objective of maximising people's opportunities for ongoing participation within society as they proceed into older age is likely to be a major challenge for policy makers in industrialized nations. Cross-country analyses (of Canada, Finland, Germany, Italy, Japan, the Netherlands, Sweden, the United Kingdom, and the United States) indicate that there is a remarkably similar pattern in the age profile of work and passive and active leisure activities across countries [32]. Despite considerable variation in hours spent doing paid work between countries for adults in the 45–54 age group, intercountry differences seem to vanish by the age of 75 years and over, with time that was previously spent doing paid work being predominantly reallocated to passive rather than active leisure activities [32]. Much of older people's time also appears to be spent at home. Research undertaken in Berlin with community-based and institutionalized adults aged 70 years and over, for example, indicates that older people spent the majority of their day alone at home (80% of awake time) and less than a fifth of their day outdoors (18.7%) [33]. Similarly, more recent Australian research indicates that only a small proportion of time is spent away from home (90.2% spent at home) by community-based adults aged 65 years and over, with the mean number of episodes spent away from home in the previous week being 6.3 (SD = 4.5; range 0–19) [34]. In addition, the average daily time spent away from home by older people who have had a stroke and those who have not appears to very similar (0.9 versus 1.1 hours, resp.) [34]. Age- and/or health-related issues are implicated, however, in a substantial difference observed in older Australians' engagement in volunteering. Twice as many current drivers (66%) as retired drivers (30%) reported involvement in this particular form activity [35].

The current study used a mixed-methods approach to explore areas of interest within the area of active aging that are currently underresearched, including (1) the connection between older people's self-perceptions of “being active” and indicators of their health and participation (domains which are proposed by the WHO to be two of the three key determinants of “active aging”); (2) older people's perceptions of characteristics of their respective communities, which are generally conceived to facilitate aging in place; and (3) the extent to which older people's out-of-home physical activity differs across different built environments, based on high quality objective measures of active travel (walking and biking).

## 2. Materials and Methods

### 2.1. Participants and Study Locations

A convenience sampling method was used to recruit a total sample of 48 adults aged 55 years and over, comprising 4 subsamples of equal numbers (*n* = 12) of individuals living in (1) inner city suburbs and (2) suburbs outside the inner city area of a capital city, (3) a regional city, and (4) a rural town in Queensland, Australia. Recruitment took place via two methods. The first involved residents of inner city suburbs of Brisbane (within 5 kms of the central business district) who had participated in a previous project (Living in the City) and also indicated their willingness to be contacted for future research being invited to participate as representatives of high density living environments. Eleven were recruited. The second recruitment method involved key community organisations and groups being approached to assist in identifying potential participants living in Brisbane suburbs situated outside the inner city, Toowoomba (a regional city), and Roma (a rural town), as well as one individual to complete the Brisbane inner city subsample. Each individual was subsequently contacted via phone and/or email and invited to participate in the study. The total sample comprised 24 males and 24 females with each of the four subsamples being comprised of equal numbers of males and females (a thirteenth individual from Roma who volunteered to participate in the study jointly with her spouse was excluded from the study to ensure both an equal gender ratio and subsample size).

All participants were ambulatory and the age range for the whole sample was 56 to 93 years (average age of 72.02 years; SD = 8.46).  Table 1 provides a demographic profile of the sample across the four study locations with respect to age, income, marital status, and current housing (derived from survey data). The population density for each of the four study locations is reported in the footnotes of the same table. The mean age for three of the subsamples was almost identical, but the mean age of the regional city (RC) subsample was between 2.5 and 2.7 years higher than the inner city (IC), city suburban (CS), and rural town (RT) groups. Nearly all of those from the IC group had high incomes, while the majority living in the CS, RC, and RT locations had incomes that fell in the low range. The most common source of income across the whole sample was the old age pension (33.3%), but superannuation also provided whole or part of the annual incomes of one-third of the sample. Only a small proportion of the whole sample was engaged in full- or part-time work (15.2%). The majority of participants in each subsample were married and also owned the homes in which they resided. All members of the IC group were living in a flat or unit (interview data revealed that most lived in a unit), while the majority of the other three subsamples were living in a house. Most of the older people in each of the four study locations owned their own dwelling.

### 2.2. Data Collection

Quantitative data were gathered from a GPS device (which captured one logged position every one minute and was used to track all out-of-home travel over seven consecutive days) as well as responses to a brief survey contained in the front section of a travel diary. The GPS device, GPS charger, and diary were posted to each participant, which they returned to the project site by post or courier at the end of the tracking period. Qualitative data were obtained from daily travel diary entries made by each participant (documenting all out-of-home travel, activities undertaken outside the environment, and the mode of transport used for each trip). In a few cases, participants forgot to either recharge their GPS device or take the device when they left home on one or two days and so were asked to continue completing their travel diaries and using the GPS to ensure that their out-of-home travel was monitored for the required seven days. Following the return of the completed diaries, GPS device, and charger, the GPS data were converted into individual time/space maps for each participant using Google Earth software. Diary entries were used to color-code each trip line shown on the maps to indicate the means of travel used for each trip made (by car, bus, ferry, train, taxi, and bicycle and on foot). These maps were subsequently used to direct discussion and verify correspondence between the GPS data and diary entries during in-depth, semistructured interviews held with each participant approximately two weeks after the tracking period. Interviews lasted approximately 90 minutes on average. Approval for this study was given by the QUT Human Research Ethics Committee.

### 2.3. Measures

#### 2.3.1. Survey Data

Survey items tapped demographic characteristics (see Table 1) as well as attributes of the built environment in which participants lived. The latter were assessed from endorsement of statements that represented reasons for them living in their current community and included proximity to destinations (it is close to shops, etc., and close to my family/friends) and safety (it is a safe area). Participants' perceptions of the age-friendliness and disability-friendliness of their communities were captured by responses to two separate items with a shared lead-in question (“Do you think your community is: (1) “age-friendly”; and (2) “disability-friendly”. Available options were the same for each item (“yes”, “no”, “do not know”, and “never thought about it”). Self-perceptions of being healthy and being active were tapped from responses to two single-item, five-level Likert scales (options and coding for these variables are reported in Table 3). Survey forms also included a question asking about all modes of transport normally used for moving around one's neighbourhood (for details of survey item and available options, see footnotes of Table 4).

#### 2.3.2. GPS and Travel Diary Data

Travel diary entries were used in conjunction with GPS data for determining the number of days when participants ventured out of home, their use of public transport, time spent travelling on foot and by bicycle outside of the home environment, and the total time spent traveling outside home over the seven-day tracking period. In combination, travel diary entries and GPS tracking produce high quality data on travel behavior [36]. A new variable was created which measured the total time spent travelling on foot and by bicycle to ensure that all of participants' travel by active means over the tracking period was captured.

The continuous variables for time spent walking and travelling by active means were converted into a six-level measure of 30-minute intervals (ranging from zero to a maximum of over 2 hours) for the purpose of correlational analysis because of the wide variation in minutes travelled on foot and walking/biking by those who walked or used active means for a period of more than two hours. Travel time captured by GPS in off-road open spaces (such as parks and golf courses) was included, but any time spent inside buildings (such as shopping centers) was excluded from travel calculations.

### 2.4. Analysis

All of the analyses were undertaken using SPSS 21.0. Cross tabulations were run for individual categorical and ordinal variables for descriptive purposes. Results are reported in tables in the form of cell counts due to the small size of each subsample, with percentages being shown in the totals column of each table. Missing values are reported in the tables (as “no response”) and are included in the row totals.

Pearson correlation analyses were performed to identify relationships between select variables for the entire sample for exploratory purposes to determine relationships between self-perceptions of being active and being healthy and objective measures of participation (number of days when participants travelled out of their homes) and travel by active means outside the home environment. Comparative analyses of the four study locations were undertaken using a nonparametric approach, the Kruskal-Wallis test of significance, due to its appropriateness for three or more independent, small, and nonrandom samples. The statistical significance of differences between groups determined by this test is based on ordinal information only, with observations being ranked and the mean rankings of the various groups being compared [37]. The original continuous variable minutes spent walking and minutes spent walking/biking were used for these analyses to ensure true ranking of data (actual minutes) and minimization of tied ranks.

## 3. Results and Discussion

### 3.1. Results

#### 3.1.1. Older People's Perceptions of Their Communities Being Age- and/or Disability-Friendly

Table 2 shows that the majority (64.6%) of the whole sample believed that their communities were “age-friendly” but less than half (41.4%) agreed that they were “disability-friendly.” Interestingly, a similar proportion (41.7% in total) neither agreed nor disagreed about the disability-friendliness of their communities and either “did not know” (18.8%), had “never thought about it” (10.4%), or did not respond to the question (12.5%). More individuals within the RC and RT subsamples rated their communities as being age- and disability-friendly than the two capital city subsamples (IC and CS).

#### 3.1.2. Perceived Attributes of the Community

Participants' reasons for living in their current community are shown in the lower part of Table 2. One-third of the whole sample identified their respective communities as being safe areas (33.3%) and similar proportions reported proximity as a factor that contributed to their reasons for living in their current communities (“close to family/friends” = 33.3%; “close to shops, etc.,” = 29.2%). More older people living in the RC location identified being close to family/friends than those in the IC, CS, and RT groups (7 versus 3) as a reason for living in their current community, while similar numbers selected “being close to shops, and so forth.” Only two older people living in the CS area agreed that safeness of the area was a reason for them living in their communities (compared to 4 or 5 in the other three groups).

#### 3.1.3. Self-Perceptions of Being Healthy and Active and Participation outside Home

The patterns of self-ratings of being active and of being healthy were each found to follow a normal distribution with a left skew (prone to being healthy and active), results that are to be expected for a community-based older sample. The majority rated themselves as being either “very active” (18.8%) or “active” (47.9%), and just under one-third of the sample considered themselves to be “somewhat active” (29.2%). Only two identified themselves with inactivity (one selected “inactive” and the other “very inactive”). A majority also perceived themselves to being either “very healthy” (20.8%) or “healthy” (45.8%). Less than one-fifth rated themselves as “unhealthy” (14.6%) and none of the participants reported being “very unhealthy” (see Table 3).

No relationship was found to exist between self-ratings of being active and self-rated health status (*r* = 0.24; *P* = 0.098). Self-perceptions of being active were also found to be unrelated to age (*r* = −0.15; *P* = 0.317), but a statistically significant, negative correlation was found between self-rated health status and age (*r* = −0.32; *P* = 0.027).

Only five of the total sample of 48 travelled outside of their home in four days or less over the monitored week. The number of days on which participants ventured out of home was positively associated with their self-ratings of being active (*r* = 0.36; *P* = 0.011) but was unrelated to both self-ratings of health (*r* = 0.11; *P* = 0.468) and age (*r* = −0.26; *P* = 0.072), and the subsample means and medians for days travelled outside home were remarkably similar (see Table 3).

#### 3.1.4. Out-of-Home Travel Using Active Means

The first part of Table 4 summarizes self-report travel-related information derived from survey to provide context to the GPS tracking results for time actually spent walking and walking/biking combined. The vast majority (81.3%) reported being drivers of a motor vehicle. Most drove cars but one person drove a motorcycle and another two drove both a car and a motorcycle. Another six (12.5%) travelled by motor vehicle as a passenger, and only three reported that they did not travel by motor vehicle (as either drivers or passengers). Less than half (41.7%) of the whole sample reported ever using public transport, and all but one of this group were from the IC and CS locations. None of the RC sample reported using public transport even though bus services are available in their location. Both the RC and RT locations only have access to train travel for the purpose of traveling outside of their local areas (rail link extends eastbound to Brisbane and westbound to Charleville), and only one participant from the RT group reported ever using the train. GPS data showed that only a quarter (25.0%) of the sample actually used public transport during the tracking period.

The actual time spent walking and time spent walking and biking combined by individuals living within the four study locations (based on GPS data) are shown in the lower section of Table 4. With respect to walking, one-third of the whole sample did not travel on foot out of their home environments at all over the tracking period. Considering this group and the next two categories (1–30 mins and 31–60 mins), more than half (56.2%) of the whole sample travelled out of home on foot for an hour or less during the monitored week. Just under one-third (31.3%) travelled on foot for more than two hours over the seven days. No correlation was found between time spent walking and self-perceptions of being active (*r* = 0.12; *P* = 0.401) and being healthy (*r* = 0.12; *P* = 0.414) or age (*r* = −0.06; *P* = 0.688).

In Table 4, it can also be seen that the number of nonwalkers in the lower density suburbs of Brisbane was twice that of their counterparts living in the inner city suburbs of Brisbane, with the numbers of nonwalkers in the IC group being similar to both the RC and RT groups. Considering the actual time spent walking (in minutes) by each individual across each of the four groups, however, the Kruskal-Wallis test indicated there was no significant difference in the mean rankings by study location (*H*(3) = 0.892; *P* = 0.827).

Once time spent traveling by bicycle was taken into account, only three individuals in the CS group were found to have spent no time using active means of travel (on foot or biking), but the remainder of the findings were similar to those observed for walking. Time spent travelling by active means was found to be unrelated to both self-ratings of being active (*r* = 0.16; *P* = 0.279) and healthy (*r* = 0.18; *P* = 0.216) and to age (*r* = −0.16; *P* = 0.284). No significant difference between the four study locations was identified in the mean rankings of active travel (based on actual minutes) either (*H*(3) = 0.520, *P* = 0.915).

**Figure 1:**
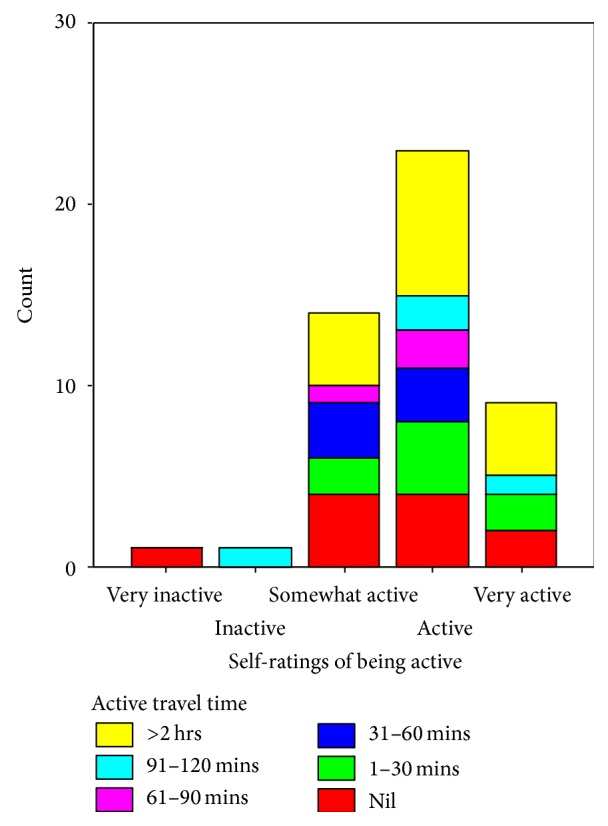
Active travel time by self-perceptions of “being active.”

**Figure 2:**
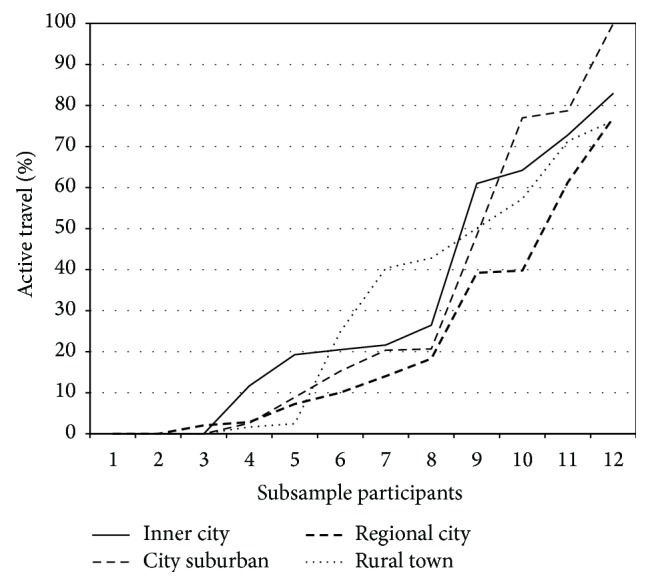
Proportion of total time travelled using active means over seven days by study location.

A visual representation of the lack of correlation between self-perceptions of being active and use of active means of travel is shown in Figure 1. Note, for example, that self-ratings of being active among those who spent no time walking or biking are distributed across four categories, from “very inactive” to “very active.” A visual representation of the proportion of all time travelled over the tracking period spent using active forms of transport is shown in Figure 2. The similarity in the overall pattern that emerged for each of the four study locations is clearly evident and the Kruskal-Wallis test confirmed that there was no significant difference in the mean rankings of the proportion of overall time spent travelling by active means across the four study locations (*H*(3) = 0.457, *P* = 0.928).

## 4. Discussion

Overall, the findings contribute to a relatively small but growing empirical literature focused on active aging, by way of whole sample analysis and equal-sized subsample comparison. They add value to this body of evidence in five main ways. Firstly, the whole sample analyses enabled determinations to be made about the distribution of self-ratings of being active among community-based adults living in Australia. Like Bowling's [7] study undertaken in the United Kingdom (based on a larger sample than the current study), the most common self-rating for being active was the fourth level of a five-level single-item Likert scale (“active” in this study and “fairly actively” in Bowling's study). However, while the second most common rating in Bowling's [7] study was the fifth-level option “very actively,” our research found the third-level option “somewhat active” to be the second most common, followed by “very active.” This difference may be attributable in part to the wording and response options used by each study but may reflect either true difference in activity levels between older people in the United Kingdom and Australia or difference in the benchmark used by older people in these two nations when making this form of self-assessment. Replication studies of Bowling's [7, 10] research are needed in Australia and elsewhere in order to further understand older people's perceptions about the key constituents of active aging from their own perspective if we are to gauge similarities and differences across different cultures and countries.

Secondly, the current study was able to investigate correlates of self-ratings of being active beyond those examined by previous studies. Our finding that age was unrelated to self-ratings of “being active” is consistent with Bowling's [7] study. Our finding that self-rated health status was unrelated to self-perceptions of being active differs however. Bowling [7] found a strong positive association between them. This inconsistency may rest on the nature of the sample used in the current study, whereby three-quarters were recruited through community organisations (and thus were actively participating in community activities) and around two-thirds reported being “healthy” or “very healthy” or differences in the measure of health status used in each study (Bowling's study used presence of a “limiting longstanding condition or disability” while this study used self-rated health status). The negative correlation we found between age and self-rated health is consistent with previous research showing an association between increasing age and more chronic health problems and declining functional ability [8].

Thirdly, this study had the capacity to explore whether self-ratings of “being active” were connected with actual behavior. The fact that these self-ratings were positively correlated with the number of days when time was spent out of home but unrelated to levels of out-of-home physical activity raises the possibility that older people in this study have embraced the idea that “active aging” is primarily about one's current participation within society. Future research efforts need to be directed at exploring the degree that self-assessments of being active as people age correspond with objective measures of both participation and outdoor physical activity. Active aging objectives are unlikely to be met if a gap exists between the meanings ascribed to active aging by older people and their actual behavior with respect to the latter. More than half of the sample (all ambulatory and mostly healthy) spent a daily average of between zero and 8.6 minutes walking outside of home over seven days. If this level of outdoor physical activity is representative of these participants' normal pattern of walking outdoors, their long-term prospects of remaining ambulatory and capable of performing daily life activities could potentially be compromised, with this in turn eventually undermining their capacity for living independently, aging in place, and participating in society. Our finding that less than one-third of older people in this study had walked for more than 2 hours over seven days is consistent with findings from studies undertaken in the United States [19, 20], where a similar car culture to Australia's prevails. Given the accumulated evidence about barriers to walking (see [16, 27]) and the difficulties associated with using public transport [14] experienced by older people, as well as this group's reliance on cars [12, 24], it may be that the developments of community-based programs that encourage walking behavior among older people with a specific focus on recreational walking in spaces where pedestrian infrastructure is in good condition and free of traffic (e.g., parks and cyclist-free pedestrian walkways) are needed. These programs could target individuals as well as groups (such as senior citizens organizations) and may require the use of motorized transport (e.g., private motor vehicles and public or private bus) for gaining access to well-maintained, safe walking tracks. This approach could potentially create opportunities for older people to overcome their fears about moving outdoors, increase their number of social interactions [17], maintain or improve their overall health and well-being through the gaining of regular exercise [18, 31], and also change expectations that an increasingly sedentary life is a natural part of the aging process [31].

Fourthly, this study was able to explore whether different built environments (with varying public transport services and population densities) were associated with higher or lower levels of active travel. While the lack of difference in time spent walking (or walking/biking combined) between locations observed in this study could potentially be due to the size of the groups in the subsample, there is no reason to suspect that this particular finding is simply spurious in nature. We were able to gather a range of information about each location, including older people's perceptions of the age- and disability-friendliness of their respective communities, their safeness and closeness to amenities and family/friends, and differences in public transport services. Interestingly, similar numbers of participants in each location identified being “close to shops, and so forth” as a reason for living in their current communities. Thus this particular feature of the environment was not one that differentiated the inner city subsample from the other three lower density areas, even though higher density areas are often touted to be places that provide greater and easier access to amenities, goods, and services than lower density areas. Previous research indicates that it is proximity to particular destinations (especially shopping malls, retail outlets, and places of employment) and not population density that is associated with the walking behavior of older people (see [15]). Despite differences in public transport services in each study location and in older residents' perceptions of the age- and disability-friendliness of their respective communities, the emergent pattern of the proportion of time traveled by active means over seven days by older people was remarkably similar. This along with the finding from the whole sample analysis that only one-third engaged in active travel for more than two hours, a proportion that is consistent with findings from large population studies of older people's walking behaviour [19, 20], raises the possibility that variability in the walking behavior of older people has more to do with normal variation within populations (as a consequence of numerous factors) than it does to features of the surrounding environment. Together with the absence of any convincing evidence that there is a direct relationship between the built environment and older people's walking behavior [15] and the many barriers identified in the extant empirical literature which deter older people from venturing out of home on foot, our study's findings give grounds for questioning any taken-for-granted assumption that older people will walk outdoors more frequently if the surrounding environment is made more “walkable.” A universal approach may need to be taken with respect to the provision of community-based walking programs for older people (discussed earlier) to ensure they are widely available, irrespective of the nature of the surrounding built environment of their homes, if a substantial increase in the outdoor physical activity of this segment of the population is to be achieved.

Fifthly, the mixed-methods approach used in this study provided the opportunity to explore connections between self-ratings of being active and objective measures of physical activity outside of the home domain via GPS tracking, as well as comparison of physical activity across four different settings that differ by population density and built environment (including public transport services). This combination of methods represents a major strength of our study. The capturing of time spent biking is an additional strength of this study, since research that relies on walking behavior alone ignores physical activity undertaken through alternative means. By instructing participants to document all modes of transport used for their out-of-home travel in combination with GPS tracking, this research avoided the circumstance where the research process itself leads participants to modify their walking behavior. This is a limitation of studies based on accelerometer-based information, the results of which do not necessarily reflect people's usual pattern of walking, because participation procedures (i.e., wearing an accelerometer) potentially prompt individuals to walk more frequently than normal for the monitored period.

Finally, further research based on objective measures of walking (as well as larger samples recruited from urban and rural settings) is needed if evidence-based determinations are to be made about the extent to which particular built environments foster greater levels of walking among older people (as proposed by Giles-Corti et al. [22]). The constraints imposed on the size of this study's sample by the nature of its methodological approach may be averted by future studies, given that technological advancements in recent years are making this approach increasingly feasible for larger samples. The developments of digital diaries, which are able to be used by participants at the same time and on the same device as GPS tracking takes place, hold great promise in their capacity to streamline data collection of movement and activity information, as well as data management and analysis, and may also reduce participant burden (see Draijer et al. [36]). Whether or not these new devices are useful for older samples (who may resist making diary entries in a technological device in place of paper and pencil diaries) remains to be seen however. The high quality of data produced by GPS/travel diary methods suggests significant benefits could accrue from the trialing of these new technologies with older samples. If successful, substantial headway could be made in expanding the body of evidence on the relationship between the built environment and older people's walking behavior gathered from high quality measures of walking.

## 5. Conclusions

Given that “active aging” is being promoted as a key policy agenda for dealing with the growing aging populations in industrialized nations, this particular subject area warrants much greater attention than it has attracted to date. It is imperative that older people understand the nature of all the constituents of the active aging concept as outlined by the WHO [1] if the objectives of its policy framework are to be met. Currently, evidence related to the meanings older people ascribe to this concept as well as the particular constituents of active aging that inform this group's self-ratings of being active is limited. More research is needed to determine if older populations are prone to connect “active aging” with “getting out and about and doing things” in the present more than they do behaviors such as walking outdoors as a means to maintain their health into the future. “Active aging” is extremely broad in scope compared to its conceptual predecessors (“successful,” “healthy,” and “productive” aging). It may therefore be necessary for messages communicated to the public under the banner of “active aging” to clearly articulate and emphasize the need for physical activity as a means to maintain health and prolong older people's participation within society. Furthermore, the flurry of research interest in the relationship between the built environment and older people's walking behavior that appears to have been sparked by the WHO's [2] Global Age-Friendly Cities publication has not been matched by similar interest in other areas pertaining to active aging. Researchers and policy makers alike need to remain mindful that the built environment is just one of many factors affecting older people's opportunities to age actively and that modifications to the built environment may be insufficient to the task of overturning older people's unwillingness to move outdoors on foot through fear of falls and injuries from traffic and poorly maintained pedestrian infrastructure, their expectations about aging, or the perceived comfort and convenience of the motor vehicle.

## Figures and Tables

**Table 1:** Demographic profile of participants by study location.

	Inner city^a^	City suburban^b^	Regional city^c^	Rural town^d^	Totals (%)
	(*n* = 12)	(*n* = 12)	(*n* = 12)	(*n* = 12)	
*Age (in years) *					
Range	56–80	57–87	68–93	59–88	N/A
Mean	72.3	72.3	75.0	72.4	N/A
*Annual income *					
Low ($40k and below)	1	9	8	8	26 (54.2)
Mid ($40k–$70k)	1	2	3	—	6 (12.5)
High ($70k and over)	7	1	—	—	8 (16.7)
No response	3	—	1	4	8 (16.7)
*Source of income *					
Wage	3	1	1	1	6 (12.5)
Superannuation	3	3	1	—	7 (14.6)
Part superannuation	—	—	1	—	1 (2.1)
Pension	4	5	7	—	16 (33.3)
Part pension	1	—	—	1	2 (4.2)
Part super/part pension	3	3	2	—	8 (16.7)
Self-funded	2	1	1	—	4 (8.3)
Self-employed	—	—	—	3	3 (6.3)
No response	—	—	1	—	1 (2.1)
*Employment *					
Full-time	2	—	1	1	4 (8.3)
Part-time	1	1	1	—	3 (6.3)
Not employed	9	10	10	10	39 (81.3)
No response	—	1	—	1	2 (4.2)
*Marital status *					
Married	7	6	9	10	32 (66.7)
Separated	—	—	—	1	1 (2.1)
Widowed	2	1	—	1	4 (8.3)
Not married	3	5	3	—	11 (22.9)
*Dwelling type *					
Flat/unit	12	2	1	1	16 (33.3)
Duplex	—	—	1	1	2 (4.2)
Townhouse	—	—	—	1	1 (2.1)
House	—	10	10	8	28 (58.3)
Room	—	—	—	1	1 (2.1)
*Owns current dwelling *					
Yes	11	9	10	11	41 (85.4)
No	1	3	2	1	7 (14.6)

^a^IC location includes six statistical local areas (SLAs) in the capital city of Brisbane (total population in 2010 = 1,067,290); average population density of the six SLAs = 4,193.6 persons per km^2^.

^b^CS location includes 12 SLAs in Brisbane; average population density (in 2010) = 1,912.7 persons per km^2^.

^c^RC location is 127 kms west of Brisbane; total population in 2010 = 131,258; population density = 236.8 persons per km^2^ (based on statistical subdivision of Toowoomba).

^d^RT location is 420 kms west of Brisbane; total population in 2010 = 7,156; population density = 96.6 persons per km^2^ (based on SLA known as Maranoa, Roma, under statistical subdivision South West in Queensland).

**Table 2:** Participants' perceptions of their respective communities.

	Inner city	City suburban	Regional city	Rural town	Totals (%)
	(*n* = 12)	(*n* = 12)	(*n* = 12)	(*n* = 12)	
*Age-friendliness of community *					
Yes	7	6	8	10	31 (64.6)
No	—	2	2	1	5 (10.4)
Do not know	—	2	2	—	4 (8.3)
Never thought about it	4	2	—	1	7 (14.6)
No response	1	—	—	—	1 (2.1)
*Disability-friendliness of community *					
Yes	3	3	6	8	20 (41.4)
No	2	4	1	1	8 (16.7)
Do not know	3	3	3	—	9 (18.8)
Never thought about it	2	1	—	2	5 (10.4)
No response	2	1	2	1	6 (12.5)
*Community features *					
It is close to my family/friends	3	3	7	3	16 (33.3)
It is close to shops, etc.	4	3	4	3	14 (29.2)
It is a safe area	5	2	4	5	16 (33.3)

**Table 3:** Self-ratings of being healthy and of being active and days ventured out of home over a seven-day period.

	Inner city (*n* = 12)	City suburban (*n* = 12)	Regional city (*n* = 12)	Rural town (*n* = 12)	Totals (%) (*N* = 48)
*Being active* ^a^					
Very inactive	1	—	—	—	1 (2.1)
Inactive	1	—	—	—	1 (2.1)
Somewhat active	3	4	4	3	14 (29.2)
Active	6	4	6	7	23 (47.9)
Very active	1	4	2	2	9 (18.8)
*Being healthy* ^b^					
Very unhealthy	—	—	—	—	—
Unhealthy	1	3	2	1	7 (14.6)
Okay	2	2	—	5	9 (18.8)
Healthy	5	4	8	5	22 (45.8)
Very healthy	4	3	2	1	10 (20.8)
*Number of days ventured out of home* ^*^					
Two	1	1	—	—	2 (4.2)
Four	1	1	—	1	3 (6.3)
Five	—	2	4	1	7 (14.6)
Six	4	2	5	4	15 (31.3)
Seven	6	6	3	6	21 (43.8)
Mean (overall mean = 6.00)	6.00	5.83	5.92	6.25	N/A
Median (overall median = 6.00)	6.50	6.50	6.00	6.50	N/A

^a^Survey item: “think about the things you do during an average week. How active would you describe yourself to be?”; coding: 1 = very inactive; 2 = inactive; 3 = somewhat active; 4 = active; 5 = very active.

^b^Survey item: “how healthy are you?”; coding: 1 = very unhealthy; 2 = healthy; 3 = okay; 4 = healthy; 5 = very healthy.

^*^Number of days ventured out of home significantly related to self-ratings of being active at *P* = <0.05.

**Table 4:** Self-reported travel modes ever used and time spent walking and using active means of travel over a seven-day period.

	Inner city	City suburban	Regional city	Rural town	Totals (%)
	(*n* = 12)	(*n* = 12)	(*n* = 12)	(*n* = 12)	
*Self-reported modes of travel ever used* ^a^					
Walking	10	8	6	10	34 (70.8)
Bicycling	1	2	—	1	4 (8.3)
Public transport	9	10	—	1	20 (41.7)
Motor vehicle					
Car as driver only	7	7	5	5	24 (50.0)
Car as driver or passenger	3	2	4	3	12 (25.0)
Car as passenger only	2	1	2	1	6 (12.5)
Driver of motorcycle	—	—	1	—	1 (2.1)
Driver of car and motorcycle	—	—	—	2	2 (4.2)
Neither driver nor passenger	—	2	—	1	3 (6.3)
*Actual travel by public transport over seven days *					
Bus	2	5	1	1^b^	9 (18.8)
Train	—	2	—	—	2 (4.2)
Ferry	3	1	N/A	N/A	4 (8.3)
Total number using public transport^c^	4	6	1	1^b^	12 (25.0)
*Time walked over seven days in categories *					
Nil	3	6	3	4	16 (33.3)
1–30 mins	—	1	3	1	5 (10.4)
31–60 mins	2	1	1	2	6 (12.5)
61–90 mins	1	1	1	—	3 (6.3)
91–120 mins	2	—	1	—	3 (6.3)
>2 hrs	4	3	3	5	15 (31.3)
*Actual minutes walked *					
Range	0–586	0–790	0–293	0–590	N/A
Mean rankings (Kruskal-Wallis test)^d^	26.38	21.92	23.46	26.25	N/A
*Time spent walking/biking over seven days *					
Nil	3	3	2	3	11 (22.9)
1–30 mins	—	2	4	2	8 (16.7)
31–60 mins	2	1	1	2	6 (12.5)
61–90 mins	1	1	1	—	3 (6.3)
91–120 mins	2	1	1	—	4 (8.3)
>2 hrs	4	4	3	5	16 (33.3)
*Actual minutes using active means of travel *					
Range	0–960	0–1562	0–293	0–590	N/A
Mean rankings (Kruskal-Wallis test)^d^	25.67	25.42	22.04	24.88	N/A
*Proportion of total time travelled by active means *					
Range	0–83.0	0–100.0	0–77.0	0–76.3	N/A
Mean rankings (Kruskal-Wallis test)^b^	26.08	24.83	22.33	24.75	N/A

^a^Survey item: “how do you get around? Tick all that apply”; options: I walk; I use a bicycle; I drive myself with a … [car, motorcycle, motored wheelchair, or mobility scooter]; someone else drives me: [my partner, my children/grandchildren, community members, social or senior services, or taxi]; I use public transport [bus, train, or ferry].

^b^Travelling by private bus, as there is no public bus service in Roma.

^c^Only 12 individuals used public transport as some used more than one mode during the tracked seven days.

^d^No statistically significant difference between groups at *P* = <0.05.
